# Anti*-Helicobacter pylori *Compounds from *Oliveria decumbens* Vent. through Urease Inhibitory *In-vitro* and *In-silico* Studies

**DOI:** 10.22037/ijpr.2021.114485.14876

**Published:** 2021

**Authors:** Mahdieh Eftekhari, Mohammad Reza Shams Ardekani, Mohsen Amin, Mahboubeh Mansourian, Mina Saeedi, Tahmineh Akbarzadeh, Mahnaz Khanavi

**Affiliations:** a *Pharmaceutical Sciences Research center, Health Institute, Kermanshah University of Medical Sciences, Kermanshah, Iran. *; b *Department of Pharmacognosy and Pharmaceutical Biotechnology, Faculty of Pharmacy, Kermanshah University of Medical Sciences, Kermanshah, Iran. *; c *Department of Pharmacognosy, Faculty of Pharmacy, Tehran University of Medical Sciences, Tehran, Iran. *; d *Department of Drug and Food Control, Faculty of Pharmacy, Tehran University of Medical Sciences, Tehran, Iran.*; e *Medicinal Plants Research Center, Yasuj University of Medical Sciences, Yasuj, Iran. *; f *Department of Pharmacology, Faculty of Medicine, Yasuj University of Medical Sciences, Yasuj, Iran. *; g *Persian Medicine and Pharmacy Research Canter, Tehran University of Medical Sciences, Tehran, Iran. *; h *Medicinal Plants Research Canter, Faculty of Pharmacy, Tehran University of Medical Sciences, Tehran, Iran. *; i *Department of Medicinal Chemistry, Faculty of Pharmacy, Tehran University of Medical Sciences, Tehran, Iran.*; j *Faculty of Land and Food Systems, University of British Columbia, Vancouver, BC, Canada.*

**Keywords:** Anti-Helicobacter pylori, Urease inhibitory, Bioactive phytochemicals, Molecular docking, Oliveria decumbens Vent

## Abstract

*Oliveria decumbens* Vent. has been used by indigenous people of southwest Iran for treating peptic ulcers and gastrointestinal infections. This study aimed to investigate the antibacterial activity of *Oliveria decumbens* extract and fractions and to analyze the bioactive components of the fractions. Total plant extract and different fractions of *Oliveria decumbens* Vent. were prepared. Antibacterial activities were evaluated against the clinical strain of *Helicobacter pylori* and standard strains of *Staphylococcus aureus*, *Staphylococcus epidermidis*, *Escherichia coli,* and *Pseudomonas aeruginosa* using agar dilution and disc diffusion methods. Phytochemical analysis of the fractions was performed using silica gel chromatography and 1D and 2D NMR spectroscopy. Moreover, the urease inhibitory effects of the isolated compounds were assessed *in-vitro* and *in-silico*. Three novel kaempferol derivatives and two thymol derivatives were isolated from *Oliveria decumbens* aerial parts, and the structures were determined by comparison with published data. The *n*-hexane fraction was found to exert the most significant anti*-H. pylori *activity with the minimum inhibitory concentration of 50 µg/mL. All fractions demonstrated antibacterial activity toward *S. aureus*. *In-vitro* urease inhibition assay showed that stigmasterol, tiliroside, and carvacrol were found to be the most potent enzyme inhibitors in the isolated compounds. Molecular interactions of the compounds with the active site of urease were supported by the molecular docking analysis. Novel bioactive compounds in *Oliveria decumbens* were described in this study. The antibacterial effects suggested the potential use of the compounds in pharmaceutical formulations inconsistent with the traditional use of the plant in the treatment of gastrointestinal infections.

## Introduction

*Helicobacter pylori* infection has been identified as the main cause of gastrointestinal disorders such as duodenal ulcer disease, peptic ulcer disease, gastric carcinoma, primary gastric B cell lymphoma, and hyperemesis gravidarum (1). The prevalence of diseases associated with *H. pylori* infection and the corresponding health care costs highlight the importance of the subject and the development of efficient therapeutic strategies based on anti-*H. pylori* agents (2).

 Urease is a nickel-containing enzyme that plays a vital role in inducing* H. pylori *infection since it provides the desired pH for surviving this pathogen. As urease is one of the virulence factors responsible for *H. pylori* colonization, urease inhibition has been a strong tool for treating *H. pylori* infection (3). 

Nowadays, medicinal plants and natural products are highly in demand around the world due to their versatile pharmaceutical and medicinal properties (4). There is a lot of research focusing on the antibacterial activity of the plants as well as isolated bioactive compounds because of failure in the treatment of infections by conventional antibiotics (5). 

 Phenolic compounds and flavonoids are abundant and ubiquitous in natural products, which have shown efficient anti-*H.* *pylori* and antibacterial properties through various mechanisms (5-7). For example, quercetin is known for the inhibition of *H. pylori* urease as well as bacterial DNA gyrase (8). According to Zhang *et al.* (2009) report, apigenin inhibits the β‐hydroxyacyl‐acyl carrier protein dehydratase from *H. pylori* (HpFabZ) (9). Kaempferol derivatives have also depicted antibacterial activity (10) and considerable inhibitory effects against the growth of *H. pylori* (11). 

The literature review shows that the plant species in the family Apiaceae are a remarkable source of flavonoids, mainly ﬂavonols and ﬂavones (12).* Oliveria decumbens* Vent. belongs to the family Apiaceae which is distributed in the west and southwest of Iran and traditionally used for the treatment of pulmonary infections and gastrointestinal diseases (13). Indigenous people of the southwest of Iran used aqueous infusion or dried powders of aerial parts of *O. decumbens* locally known as “Moshkoorak” to treat peptic ulcer and gastrointestinal infections and relieve thirst in children (14). 

 Previous studies revealed that *O. decumbens* essential oil possessed antibacterial and antifungal activities because of a high percentage of thymol and carvacrol (15). A previous study revealed that hydroalcoholic extract of* O. decumbens *demonstrated remarkable antibacterial activity against methicillin- and cefixime-resistant* Staphylococcus aureus* strains(16). Although there are several reports on the biological properties of the plant, no complementary phytochemical studies have been achieved, and they usually are limited to essential oil analysis (15). 

In the present study, we were focusing on the probable anti-*H. pylori* activity of *O. decumbens *according to traditional medicine and available studies, anti-*H. pylori*, urease inhibitory, and antibacterial activities of different fractions of *O. decumbens* aerial parts were evaluated. Also, phytochemical analysis of aerial parts of *O. decumbens* was conducted, and isolated compounds were a candidate to be evaluated against urease inhibition. Finally, molecular docking was performed to recognize possible interaction modes between those compounds and urease active sites.

## Experimental

*General procedures*

All solvents and materials were analytical grades. Silica gel with different mesh (Merck), Sephadex LH-20 (Fluka, BioChemika), and RP-18 (Fluka) was used for column chromatography. TLC aluminum sheets (Si gel 60 F254 and RP_18 F254) (Merck) were used for monitoring of spots under UV. ^1^HNMR and ^13^CNMR spectra were recorded on a Bruker NMR 500 spectrometer (Bruker, Germany). Chemical shifts (δ) were expressed in ppm using tetramethylsilane (TMS) as the internal standard, and coupling constants were calculated (*J*) in Hz.

*Collection and authentication of plant*

The aerial parts of *O. decumbens* Vent. were collected in June 2014 from Kohgiluyeh and Boyer Ahmad province, Iran. The sample was authenticated and deposited in the Central Herbarium of Tehran University, Tehran, Iran (Voucher specimen: 451500 TUH).

*Extraction and fractionation*

Dried and finely powdered of the plant (1500 g) were extracted by methanol/distilled water (80/20) at room temperature three times using the maceration method. After removal of the solvent under vacuum at 40 ºC, the residue known as total extract was subjected to liquid-liquid extraction by different solvents (*n*-hexane, chloroform, and ethyl acetate). The residue left after fractionation was solved in methanol and filtered off to remove the solid. In each step, the solvent was evaporated, and the concentrates were lyophilized. 

*Evaluation of anti-Helicobacter pylori activity*

Anti-*Helicobacter pylori *activity of the samples was investigated by an agar dilution method according to our previous study (17). The classic Clinical and Laboratory Standards Institute methods use broth microdilution only for conventional antibiotics, and modifications of those methods are suggested for other substances such as natural health products (NHPs). The Agar dilution method has been standardized previously as an appropriate method to determine minimum inhibitory concentrations (MIC) of water-insoluble and colored plant extracts. The Agar dilution method was performed in Brucella agar plates supplemented with 20% fetal bovine serum (FBS, Gibco, USA) containing different concentrations of the plant extract or fractions ranging from 50 to 2000 μg/mL. A volume of 100 μL standardized *H. pylori *inoculum strain RIGLD-HC180 obtained from antral biopsy samples of patients at Taleghani Hospital, Tehran, Iran (equal to 5 × 10^5^ cfu) was spread onto the plates and incubated for 72 h at a microaerophilic condition in a jar containing Anaerocult^®^ C (Merck, Germany) at 37 °C. Growth control plates were included in each experiment. The lowest concentrations of the total extract or different fractions that inhibited visible bacterial growth (containing no colonies) were expressed as minimum inhibitory concentrations (MICs). All the experiments were performed in triplicate.

Plates, including amoxicillin, were used as a positive control (17).

*Evaluation* *of antibacterial activity*

Antibacterial activity of total extract and different fractions were examined by a disc diffusion method based on the standard CLSI M100 against two Gram-positive bacteria, *Staphylococcus aureus *ATCC 6538, and *Staphylococcus epidermidis* ATCC 12229 and two Gram-negative bacteria, *Escherichia coli *ATCC 8739 and* Pseudomonas* *aeruginosa* ATCC 9027 as previously reported (17). Briefly, overnight cultures of bacteria were used to prepare a bacterial inoculum in sterile normal saline. The bacterial inoculum was standardized to the turbidity of 0.5 McFarland (the density equivalent to 1.5 × 10^8^ cfu/mL). The surface of Mueller-Hinton (MH) agar plates was covered by 100 µL of diluted bacterial suspension containing 5 ×10^5^ cfu. Different concentrations of the plant extract or fraction were prepared. Sterile blank discs (6.4 mm diameter) containing different concentrations of the plant extract or fractions (15 μL) were placed on the agar plates. The mean inhibition zone diameter was measured after overnight incubation at 37 °C. Ciprofloxacin and penicillin discs were used as positive controls (17).

*Isolation and purification*

*n*-Hexane, ethyl acetate, and methanol fractions were selected for phytochemical investigations. *n*-Hexane fraction (5.7 g) was placed on silica gel (30-70 mesh, 5 × 50 cm) and eluted with a gradient mixture of EtOAc/*n-*hexane (0:10 to 10:0) obtained six fractions (**1-6**). Fraction** 1** afforded purified compound** 1 **as a colorless oil (30 mg). Fraction** 2** was subjected to silica gel (70-230 mesh, 1.5 × 50 cm), and four fractions were obtained (2a-2d). Fraction** 2b** was subjected to Sephadex LH-20 to obtain compounds 2 and 3 (red oil, 40 mg) mixture. Moreover, compound 4 (white crystals, 25 mg) were afforded from fraction** 2c**. 

 The ethyl acetate fraction (1 g) was chromatographed on a column of silica gel (30-70 mesh, 1.5 × 50 cm) eluted with a gradient mixture of EtOAc /*n-*hexane (0:10 to 10:0) to yield ten fractions (**1-10**). Fractions** 4** and **5** were further separated on a Sephadex LH-20 column to give compound** 5** (22.8 mg, R_f _= 0.6 using ethyl acetate/methanol (9:1)) and compound** 6** (33.2 mg, R_f _= 0.8 using ethyl acetate/methanol (9:1)). Both compounds **5 **and **6** were obtained as yellow crystals, which were observed as black spots under UV light at 254 on a TLC sheet. Moreover, these spots were shown in yellow when sprayed with *p*-anisaldehyde-sulfuric acid and then heated.

Fraction** 6** was subjected to Sephadex LH-20, and four fractions (**6a-6d**) were yielded. Fraction **6c** was moved to reverse-phase silica to obtain the mixture of compounds **7** and **8** (86 mg, amorphous powder, R_f_ = 0.5 using ethyl acetate/methanol (9:1) system). Those corresponding spots were black under UV light at 254 and were appeared as purple spots when sprayed with *p*-anisaldehyde-sulfuric acid.

Furthermore, methanol fraction (10 g) was moved on a vacuum column chromatography (VLC) (silica gel 30-70 mesh, 10× 15 cm) eluted with a gradient mixture of MeOH/ CHCl_3_ (0:10 to 10:0) to yield seven fraction (1-7). Fraction** 4** was further separated using Sephadex LH-20 to obtain compound** 9** (22.2 mg, R_f_ = 0.77 using quaternary solvent system, ethyl acetate/water/acetic acid/formic acid (100:26:11:11). The compound was obtained as yellow crystals, which were observed as a black spot under UV light at 254 on a TLC sheet and a yellow spot after spraying with the reagent (*p*-anisaldehyde-sulfuric acid) and subsequently heating.

The structure of all compounds was identified by a spectroscopic analysis comparing with those reported in the literature.

*Evaluation of urease inhibitory activity*

Urease inhibitory activity was evaluated based on Tanaka et al. report (18). Jack bean urease (Sigma-Aldrich, Germany) was used as a surrogate for *H. pylori* urease. Each well of a 96-well plate contained 25 μL jack bean urease (6U, Sigma-Aldrich) and 25 μL different concentrations of tested materials that were prepared in phosphate buffer (PBS, pH 6.8 ) and DMSO 1% as a co-solvent. After 30 min incubation at room temperature, 200 μL of a stock solution composed of PBS (100 mM), urea (150 mM), and phenol red were added to each well. The absorbance changes were measured at 570 nm by a microplate reader. Hydroxyurea was used as the positive control (18). 

*Molecular docking of isolated compounds* *from* *O. decumbens* *on the urease active site*

To get insight into possible interaction modes between active sites of jack bean urease and isolated compounds from *O. decumbens*, the molecular docking study was performed using Autodock software version 4.2 (19). A three-dimensional (3D) X-ray crystal structure of urease (E.C. 3.5.1.5) with 4H9M PDB code was obtained from Protein Data Bank (PDB) at the 1.52 Å resolution of Jack bean (*Canavalia ensiformis)* (20). The 2D structures of the chemical molecules were sketched and optimized using HyperChem software version 7 (Hypercube, Inc., Gainesville, FL, USA; http://www.hyper.com) as previously described (21). The canter of the grid box was positioned on the centroids of the residues, including Kcx490, His492, His519, His545, Asp633, Ni901, and Ni902 at the coordinates X = 19.067, Y = -56.327, Z = -21.334 (22). The Lamarckian genetic algorithm (LGA) as the most effective method was considered (23). For conventional systems, AutoDock was run several times (here 200 times) as previously described. Among the various conformations obtained from the docking study for each compound, the conformation with the lowest binding free energy was selected for more assessment of binding mode (24). The resulting 2D poses were analyzed visually to understand the interaction pattern by the Ligplot software (25).

## Results

*Compounds isolation and identification*

**Figure 1:**
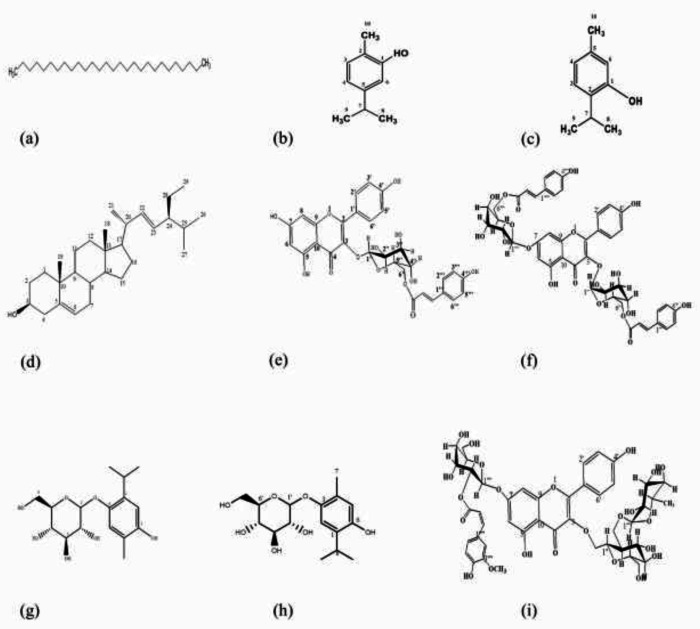
Structure of the isolated compounds from different fractions of *Oliveria decumbens *Vent. (a) Compound **1**: Octacosane, (b)Compound **2**: Carvacrol, (c) Compound **3**: Thymol, (d) Compound **4**: Stigmasterol, (e) Compound **5**: Kaempferol-3-O-(6''-O-trans-coumaryl) glucoside (Tiliroside), (f) Compound **6**: Kaempferol 3 -O-(6’’-O-trans-coumaryl)glucoside 7-O-(6’’’-O-trans-coumaryl) glucoside, (g) Compound **7**: 3-Hydroxythymol-6-O-D-Glucopyranoside, (h) Compound **8**: 6- Hydroxythymol-3-O-D-Glucopyranoside, (i) Compound **9**: Kaempferol 3-O-neohesperidoside-7-O-[2-O-(cis-feruloyl)]-D-glucopyranoside

The structure of isolated and identified compounds from *O. decumbens* was depicted in Figure 1 (**1-9**). 

Compound** 1** was obtained as a colorless oil from *the n*-hexane fraction. Based on NMR spectrum (Figure S1a, in supplementary file) and ESI-MS analysis (Figure S1b) and compared with available data (26, 27), compound** 1** was identified as octacosane (C_28_H_58_).

Compounds** 2** and **3** were mixed together. According to the NMR spectrum of the mixture (Figure S2) in comparison with available data as well as TLC analysis comparing with available standards, it was identified to be the mixture of carvacrol and thymol (28, 29).

Compound** 4** was obtained as white crystals, identified as stigmasterol based on NMR spectra (Figure S3), and compared with available literature (30).

Compound** 5** was obtained as yellow crystals and identified as 3‐*O*‐β‐‐(6″‐*O*‐coumaroyl)glucopyranoside (tiliroside) according to ^13^H- and ^13^CNMR spectra as well as available data (31-33). ^1^HNMR (500 MHz, DMSO-*d*_6_): 8.02 (2H, d, *J* = 8.5 Hz, H-2’, H-6’), 7.59 (d, 1H, *J *= 16.0 Hz, H- γ), 7.54 ( 2H, d, *J *= 8.0 Hz, H-2’’, H-6’’), 6.89 (2H, d, *J* = 8.5Hz, H-3’, H-5’), 6.79 (2H, d, *J* = 8.0 Hz, H-3’’, H-5’’),6.40 (1H, d, *J* = 16.0 Hz, H-β), 6.40 ( 1H, s, H-8), 6.17 ( 1H, s, H-6), 5.75 ( 1H, d, *J* = 7.5 Hz, H-1’’), 3.35- 4.87 (6H, m, the rest of sugars protons). ^13^CNMR (125 MHz, DMSO-*d*_6_): 177.1 (C-4), 165.8 (C- α), 164.5 (C-7), 161.1 (C-5), 160.0 (C-4’’’), 159.8 (C-4’), 156.3 (C-2), 156.1 (C-9), 144.9 (C-γ), 132.5 (C-3), 130.7 (C-2’’’/C-6’’’), 130.3 (C-2’/C-6’), 125.1 (C-1’), 120.7 (C-1’’’), 115.8 (C- β), 115.1 (C-3’/C-5’) , 114.3 (C-3’’’/C-5’’’), 103.8 (C-10), 98.7 (C-1’’), 98.1 (C-6), 93.7 (C-8), 77.7 (C-2’’), 74.1 (C-3’’), 70.1(C-4’’), 60.6 (C-5’’), 48.9 (C-6’’)( Figures S4 & S5). 

Compound** 6** was obtained as yellow crystals. According to the following NMR characterization and comparing those data with the literature (34) it was identified as kaempferol-3-O-(6’’-O-trans-coumaroyl) glucopyranoside 7-O- 6’’’’’ coumaroyl glucopyranoside. ^1^HNMR (500 MHz, DMSO-*d*_6_): 8.05 (2H, d, *J* = 8.0 Hz, H-2’, H-6’), 7.97 (2H, d, *J* = 8.0 Hz, H-2’’, H-6’’), 7.62 (d, 1H, *J *= 15.0 Hz, H- γ), 7.55 (2H, d, *J *= 8.0 Hz, H-2’’’, H-6’’’’), 7.36 (d, 1H, *J *= 15.0 Hz, H- γ´), 6.93 (2H, d, *J* = 8.0 Hz, H-3’, H-5’), 6.87 (2H, d, *J* = 8.0 Hz, H3’’’, H-5’’’’ ),6.80 (2H, d, *J* = 8.0 Hz, H3’’’’, H-5’’’), 6.42 (1H, s, H-8 ), 6.40 (1H, d, *J *= 15.0 Hz, H-β ), 6.17 ( 1H, d, *J* = 15.0 Hz, H-β´), 6.13 ( 1H, s, H-6), 5.84 (1H, d, *J *= 7.5 Hz, H-1’’’), 5.74 ( 1H, d, *J* = 7.5 Hz, H-1’’), 3.35- 4.87 (the rest of sugars protons). ^13^C NMR** (**125 MHz, DMSO-*d*_6_**)**: 177.1 (C-4), 166.1 (C-α), 165.8 (C-α´), 165.7 (C-7), 165.0 (C-4’’’’’), 164.3 (C-4’’’), 160.1 (C-5), 159.9 (C-4’), 159.9 (C-2), 156.4 (C-9), 145.2 (C- γ), 144.6 (C-γ´), 132.5 (C-3), 131.3 (C-2’’’, C-6’’’), 130.9 (C-2’, C-6’), 130.3 (C-2’’’’’, C-6’’’’’), 125.1 (C-1’), 125.0 (C-1’’’), 124.9 (C-1’’’’), 115.8 (C-β), 115.2 (C-β´), 115.1 ( C-3’, C-5’) , 114.3 (C-3’’’, C-5’’’), 113.8 (C-3’’’’’, C-5’’’’’), 103.9 (C-1’’’’), 103.8 (C-1’’), 98.3 (C-10), 98.2 (C-6), 93.7 (C-8), 75.2 (C-2’’’’), 74.6 (C-2’’), 74.3 (C-3’’’’), 73.9 (C-3’’), 71.9 (C-4’’’’), 71.4 (C-4’’), 71.0 (C-5’’’’’), 70.2 (C-5’’), 62.8 (C-6’’’’), 60.5 (C-6’’). ^1^H-^1^H COSY spectrum showed connectivities among H-2’ and H-3’ as well as H-5’ and H-6’ on the B ring of kaempferol. Also, the correlation between H-2’’’ and H-3’’’, H-5’’’ and H-6’’’, H-2’’’’’ and H-3’’’’’, and H-5’’’’’’ and H-6’’’’’ on the aromatic rings of coumaroyl moieties attached to glucopyranoside group at C-3 and C-7 of kaempferol, respectively was demonstrated. It should be noted that desired correlations between olefinic protons (β /γ and β’ /γ ‘) of coumaroyl moieties were observed. Major NOSEY correlations showed that B ring protons of kaempferol correlated with protons of aromatic ring of cumaroyl moiety attached to glucopyranoside at C-3. In addition, there was a correlation between olefinic protons (β’/γ’) coumaroyl moiety attached to glucopyranoside at C-7 and H-8 (Figures S6-S9). 

Compound** 7** was identified as 3-hydroxythymol-6-*O*-glucopyranoside according to data from NMR spectra and available literatures (35, 36). ^1^HNMR (500 MHz, DMSO-*d*_6_): 7.29 (1H, s, H-5), 6.93 (1H, s, H-2), 4.94 (1H, d, *J* = 7.0 Hz, H-1´), 3.85-3.99 ( 1H, m, H-8), 3.35-4.11 (6 H, m, H-2´- 6’ ), 2.5 (3H, s, H-7), 1.53 (3H, d, *J* = 6.8 Hz, H-9), 1.53 (3H, d, *J* = 6.8 Hz, H-10). ^13^C NMR (125 MHz, DMSO-*d*_6_): 148.9 (C-6), 147.4 (C-3), 132.1 (C-4), 121.3 (C-1), 119.1 (C-2), 111.8 (C-5), 102.8 (C-1’), 77.0 (C-2’), 76.9 (C-3’), 73.5 (C-4’), 70.1 (C-5’), 48.7 (C-6’), 25.6 (C-8), 23.1 (C-9), 22.6 (C-10), 15.8 (C-7) (Figures S10 and S11).

Compound** 8** was identified as 6-hydroxythymol-3-*O*-glucopyranoside based on data obtained from NMR spectra and literatures (35, 37). ^1^HNMR (500 MHz, DMSO-*d*_6_): 7.20 (1H, s, H-5), 7.00 (1H, s, H-2), 4.94 (1H, d, *J *= 7.0 Hz, H-1’), 3.85-3.99 (1H, m, H-8), 3.35-4.11 (6H, m, H-2’- 6’), 2.50 (3H, s, H-7), 1.53 (3H, d, *J* = 6.8 Hz, H-9), 1.53 (3H, d, *J* = 6.8 Hz, H-10). ^13^C NMR (125 MHz, DMSO-*d*_6_): 150.4 (C-6), 148.8 (C-3), 136.0 (C-4), 124.9 (C-1), 116.6 (C-2), 114.6 (C-5), 103.0 (C-1’), 77.0 (C-2’), 76.9 (C-3’), 73.6 (C-4’), 73.2 (C-5’), 60.9 (C-6’), 26.2 (C-8), 23.3 (C-9), 22.7 (C-10), 15.8 (C-7) (Figures S10 and S11). 

Compound** 9** was yield as yellow crystals. According to ^1^HNMR and ^13^CNMR spectra as well as data obtaining from literature, it was identified as kaempferol 3-Oβ -O-neohesperidoside-7-O-[2-O-(cis-feruloyl)] β -D-glucopyranoside (34, 38, 39). ^1^HNMR (500 MHz, DMSO-*d*_6_): 7.98 (2H, d, *J* = 8.5 Hz, H-2’, H-6’ ), 7.85 ( 1H, s, H-2’’’’’), 7.52 (1H, d, *J *= 11.0 Hz, H-γ), 6.91 (2H, d, *J* = 8.5 Hz, H-3’, H-5’), 6.87 ( 1H, d, *J *= 8.0 Hz, H-6’’’’’ ), 6.84 (1H, d, *J *= 8.0 Hz, H-5’’’’’ ), 6.42 (1H, s, H-8 ), 6.39, (1H, d, *J *= 11.0 Hz, H-β), 6.20 (1H, s, H-6), 5.44 (1H, d, *J *= 7.0 Hz, H-1’’), 5.42 (1H, d, *J* = 7.5 Hz, H-1’’’’), 5.08 (1H, bs, H-1’’’), 3.20-4.41 (the rest of sugars protons), 3.83 (3H, s, H-3’’’’’), 0.97 (3H, d, *J *= 6.0 Hz, H-6’’’),.

^13^C NMR (125 MHz, DMSO-*d*_6_): 177.3 (C-4), 164.5 (C- α), 161.2 (C-7), 160.0 (C-5), 156.5 (C-4’), 156.4 (C-9), 149.4 (C-2), 148.4 (C-3’’’’’), 146.9 (C-4’’’’’), 144.7 (C-γ), 133.2 (C-3), 130.9 (C-6’’’’’133.0 (C-2’ , C-6’), 122.2 (C-1’’’’’), ) 121.0 (C-1’), 115.2 ( C-3’, C-5’), 115.1(C- β), 114.3 (C-5’’’’’), 113.3 (C-2’’’’’), 103.9 (C-10),101.2 (C-1’’’), 100.9 (C-1’’), 100.7 ( C-1’’’’), 98.8 (C-6), 93.8 (C-8), 76.4 (C-3’’’’), 75.9 (C-2’’), 75.7 (C-3’’), 74.3 (C-5’’), 74.1 (C-5’’’’), 71.7 (C-4’’’), 70.9 (C-2’’’’ ), 70.3 (C-2’’’), 70.2 (C-3’’’), 70.1 (C-4’’), 68.3 (C-5’’’), 68.2(C- 4’’’’), 66.9 (C-6’’), 66.8 (C-6’’’’ ), 55.6 (C3’’’’’). 17.7 (C-6’’’’) (Figures S12 and S13).

*Anti-Helicobacter pylori and antibacterial activity*

Anti*-Helicobacter pylori* activity of total extract and different fractions were assessed using different concentrations in an agar dilution method (Table 1)*. *As shown in Table 1, the *n*-hexane fraction depicted the most significant anti*-H. pylori *activity with the MIC value of 50 µg/mL. However, the other fractions showed much lower activity against *H. pylori* (MICs = 1000, 1500, and 1750 µg/mL, respectively), and the total extract showed no inhibitory activity even at the concentration of 2000 µg/mL.

As reported in Table 2, all fractions demonstrated antibacterial activity toward *S. aureus* while all of them showed no antibacterial activity against *P. aeruginosa* even at a concentration up to 500 µg/mL. It should be noted that the total extract and all fractions except ethyl acetate fraction depicted no antibacterial activity toward *S. epidermidis* and *E. coli *in the tested concentration range. 

*Urease inhibitory*

The inhibitory activity of total extract and different fractions toward jack bean urease was reported in Table 3. The best inhibitory activity was demonstrated by n-hexane (IC_50 _= 285.44 µg/mL) and ethyl acetate (IC_50 _= 285.06 µg/mL) fractions as compared to hydroxyurea as a positive control (IC_50 _= 59.51 µg/mL). Chloroform and methanol fractions showed lower urease inhibitory activity with IC_50 _values of 375.36 and 709.42 µg/mL, respectively. However, the total extract showed no inhibitory activity. 

Moreover, the urease inhibitory of the isolated compounds as IC50 (mM) was evaluated (Table 4)*. *Among them, stigmasterol, 3‐*O*‐β‐‐(6″‐*O*‐coumaroyl), glucopyranoside (tiliroside), and carvacrol were found to be the most active as compared to positive control. It should be noted that thymol showed lower activity (IC50 = 1.33 mM) than its isomer carvacrol (IC50 = 0.70 mM). The mixture of hydroxyl thymol glucosides and compound** 9** showed similar anti-urease activity (Inhibition percentage at 100 (µg/mL) concentration = 24.6%), and no activity was induced by compound** 6** up to 250 µg/mL.

*Molecular docking of the isolated compounds in the binding site of urease*

**Figure 2:**
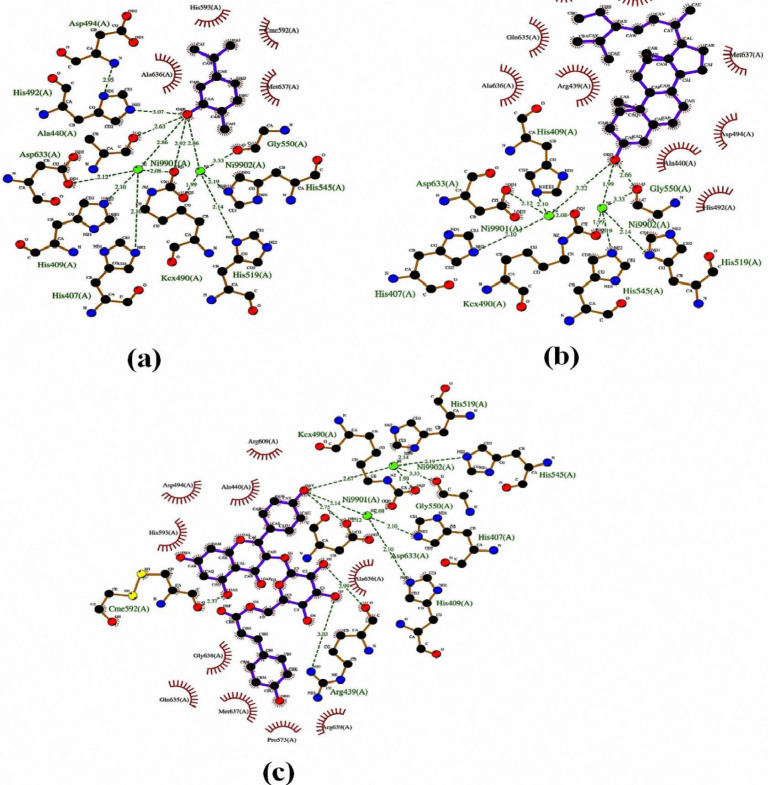
Schematic interaction of the best docking resulting from AutoDock software presented by LigPlot software for (a) Carvacrol, (b) Stigmasterol and (c) Tiliroside. In this figure, the compound exposure is highlighted in blue. Hydrogen bonding is in green and van der Waals interactions are in red circulars

Table 5 summarized all details related to the docking study of the isolated compounds in the binding site of urease. Stigmasterol, tiliroside, and carvacrol as the most active compounds inhibited the catalytic activities of urease through hydrogen bonding, metal/ion contact with Ni ions, and hydrophobic interactions. The interaction of the best-docked conformation of the ligand with active site residues is depicted in Figures 2 and S14-S18. 

The hydroxyl groups of thymol and carvacrol interacted with the Ni ion and imidazole ring of His492 (Table 5, Figure S14). In addition, the docked pose of carvacrol tightly engaged in nickel chelation along with several hydrogen bonds within the binding cavity (Figure 2a). The hydroxyl group of cyclohexyl moiety of stigmasterol highlighted the formation of a bidentate bond with two-nickel ions (1.92 Å, 3.22 Å) and Gly550 (2.66 Å) (Table 5). The substituted rings are deeply embedded into the hydrophobic pocket of the Ile411, Arg439, Ala440, His492, Asp494, Cme592, Gln635, Ala636, and Met637 (Figure 2b).

Figure 2c shows that the hydroxyl groups of kaempferol moiety in the tiliroside were also engaged in the binding cavity through nickel ions (2.14 Å, 2.67 Å) hydrogen bonds with the oxygen atom of Asp633 and Cme592 side chains. In addition, both OH groups of glucoside moiety mediated through two hydrogen bonds with the side chain of Arg439. The lowest free binding energy of tiliroside was -8.49 kcal/mol with K_i_ of 598.78 nM. This is in good accordance with the experimentally observed value of urease inhibition percentage.

Similarly, compound** 7** with glucopyranoside group at position 6 showed slightly better results than its counterpart (compound** 8**). The different OH groups of compound** 7** formed hydrogen bonding and polar bonds with Arg439, Ala440, Kcx490, His492, Asp494, Gly550, Arg609, Asp633, and nickel ions (Table 5, Figure S15). As shown in figure S16, the non-glucopyranoside moiety of compound 8 was observed more active than its homolog (compound 7) by interacting with Ni ions, Kcx490, His492, and His519. Four additional hydrogen bonds were also observed between oxygen’s glucopyranoside of compound** 8** and the key residues, including Asp494, Cme592, and Ala636 (Table 5). Compounds** 6** and **9** have interacted with other residues shown in Table 5 and Figures S17- S18.

## Discussion

The data obtained from phytochemical analysis of the aerial parts of *O. decumbens* confirmed the presence of the acylated kaempferol glycosides in the ethyl acetate and methanol fractions which were first isolated from *O. decumbens. *In the previous studies, acylated kaempferol glycosides were isolated and identified from two genera* Foeniculum *and *Eryngium, *belonging to the family Apiaceae (40, 41). Also, two thymol glucosides (compounds** 7 **and** 8**) isolated from ethyl acetate fraction of *O. decumbens* were previously reported to be isolated from *Carum ajowan* as a genus of family Apiaceae (35). 

Totally, ethyl acetate fraction showed desired antibacterial activity, which can be associated with the presence of kaempferol glycosides (tiliroside) and various phenolic compounds in this fraction. Falcão‐Silva *et al.*, reported that combination of isolated tiliroside from *Herissantia tiubae* (Malvaceae) with antibiotics such as norfloxacin, ofloxacin and ciprofloxacin led to modulated antibacterial activity and much lower MIC values (2-16 fold). It was found that the tiliroside played a significant role as a putative efflux pump inhibitor in bacteria due to the lipophilicity of the aglycon moiety (42). Also, Liang *et al.* showed that thymol derivatives such as hydroxythymol and thymol-*β*-glucopyranoside demonstrated antibacterial effect (43). It seems that thymol and kaempferol glycosides derivatives are responsible for inducing antibacterial activity of ethyl acetate fraction, which can be endorsed by the study reported by Nostro et al. on the effect of thymol on biofilm-grown *S. epidermidis* and *S. aureus* strains which emphasized the favorable activity of thymol as a lead scaffold for the desired antibacterial activity (44).

On the other hand, the *n*-hexane fraction of *O. decumbens* was the most active fraction against *H. pylori* that is probably due to the presence of stigmasterol and carvacrol as the most active anti-urease compounds. Among the isolated compounds, stigmasterol as one of the active compounds of the series (Table 5), with the lowest free binding energy -8.75 kcal/mol and K_i _= 386.65 nM showed appropriate docking score and suitable interactions in agreement with the biological activity, suggesting that this compound could be an efficient inhibitor. Furthermore, Vazirzadeh *et al.* 2019 showed that the administration of different derivatives of *O. decumbens* had remarkable antibacterial activity against streptococcus and also enhanced antioxidant status and post-challenge immunity in Nile tilapia (45). Carvacrol adopted a similar orientation to thymol. The potency of carvacrol as the most active component (Table 4) might be attributed to the hydrogen bonding interactions between the hydroxyl group and Ala440, Kcx490, His492, Ni ions (Figure 2a). In consist with our findings, previous studies revealed that carvacrol showed anti-*H. pylori* activity is more significant than thymol (17, 46).

Moreover, similar to our findings, Hřibová et al. 2014, showed tiliroside inhibits urease with a docking score equal to ‐8.9 kcal/M *in-silico* study (46).

However, the binding mode of compounds** 7** and **8** are in an almost similar way which has been investigated by the mixture of both combinations in inhibition of catalytic activities of the studied enzyme (Table 4).

The inactivity of compound** 6** was clarified by the investigation of the binding mechanism. It is obvious that the inactivity of this compound largely depends on the bulky substitution in which caused poor interaction and steric clashes. The steric hindrance of groups on adjacent positions might be responsible for the contrast behavior of compound** 6**. This created a complete shift in the conformation and ultimately prevented interactions with the key active site residues and Ni ions (Figure S17). While in the case of tiliroside, possessing a lower number of substitutions is well oriented within the binding cavity in comparison to compound** 6**. Then, the importance of hydrogen bonding interaction with key residues and metal/ionic contact with Ni is also conﬁrmed by experimental results. In addition, compounds** 6** and **9** bearing bulky substitutions were found to be lower effective than tiliroside. The OH group of the neohesperidoside moiety of compound** 9** mediated bidentate interactions with Ni ions as shown in Table 5, while the kaempferol part of the compound** 9 **presented an inert behavior which may be attributed to the spatial hindrance of some groups, resulting in moderate inhibition (Figure S18, Table 4). From the above discussion, it can be concluded that electron-rich hydroxyl groups played vital roles in the urease inhibitory activity. The hydroxyl groups of rings of all compounds except compound** 6 **interacted with crucial residues and Ni ions. To sum up, all desired interactions of stigmasterol, tiliroside and carvacrol with key residues of the jack bean urease (7, 20 and 47) were completely in accordance with those data from *in-vitro* urease inhibitory activity assay.

**Table 1:** Total extract and different fractions of *Oliveria decumbens *Vent.: Minimum inhibitory concentrations (MIC) for the growth of *Helicobacter pylori *by the agar dilution method

**Sample**	**MIC (µg/mL)**
Total extract	> 2000
Hexane fraction	50
Chloroform fraction	1500
Ethyl acetate fraction	1000
Methanol fraction	1750
Amoxicillin as positive control	0.5

**Table 2:** Inhibition zone (mm) of total extract and different fractions of *Oliveria decumbens* Vent. and antibiotic discs against some pathogenic bacteria by the disc diffusion method

Sample	*S. aureus*	*S. epidermidis*	*E. coli*	*P. aeruginosa*
Total extract (500 µg/disc)	12 mm	NZ	NZ	NZ
Hexane fraction (500 µg/disc)	8 mm	NZ	NZ	NZ
Chloroform fraction (500 µg/disc)	12 mm	NZ	NZ	NZ
Ethyl acetate fraction (500 µg/disc)	20 mm	9 mm	10 mm	NZ
Methanol fraction (500 µg/disc)	16 mm	NZ	NZ	NZ
Ciprofloxacin (5 µg/disc)	24 mm	36 mm	33 mm	32 mm
Penicillin (10 µg/disc)	24 mm	26 mm	10	-

NZ= No inhibition zone was observed.

**Table 3:** Urease inhibitory of total extract and different fractions of* Oliveria decumbens *Vent. as IC50 (µg /mL).

**Sample**	**IC50** **(µg/mL)** ± SD
Total	> 1000
Hexane	285.44 ± 0.01
Chloroform	375.36 ± 0.02
Ethyl acetate	285.06 ± 0.02
Methanol	709.42 ± 0.03
Hydroxyurea	59.51 ± 0.01

**Table 4:** **Urease inhibitory of the isolated compounds of** ***Oliveria decumbens*** **aerial parts as** **IC50 **(mM)

**Compounds**	**IC50 **(mM) ± SD
Thymol	1.33 ± 0.06
Carvacrol	0.70 ± 0.01
Stigmasterol	± 0.010.27
Tiliroside	0.45± 0.05
The mixture of hydroxyl thymol glucoside compounds	*
Kaempferol-3-O-(6''-O-trans-coumaroyl) glucopyranoside 7-O- 6''''' coumaroyl glucopyranoside	N
Kaempferol 3-Oβ -O-neohesperidoside-7-O-[2-O-(cis-feruloyl)] β -D-glucopyranoside	*
Hydroxyurea	0.78± 0.01

*= Inhibition percentage at 100 (µg/mL) concentration was 24.6 ± 0.01%

N= No inhibition was observed.

**Table 5:** Energy-based interactions detail of the identified structures isolated from *Oliveria decumbens* as the urease inhibitors

Ligand No	Docking Score (kcal/mol)	Estimated Inhibition Constant (K_i_)	Interactions detail			Distance (Å)
			Ligand	Receptor		
Thymol (Compound 3)	-4.44	553.48 µM	OAK OAK	His492 Ni902	NE2 Ni	2.80 2.64
Carvacrol (Compound 2)	-4.88	263.60 µM	OAK OAK OAK OAK OAK	Ala440 Kcx490 His492 Ni901 Ni902	O OQ1 NE2 Ni Ni	2.63 2.92 3.07 2.86 2.86
Stigmasterol (Compound 4)	-8.75	386.65 nM	OBD OBD OBD	Gly550 Ni901 Ni902	O Ni Ni	2.66 3.22 1.99
Tiliroside (Compound 5)	-8.49	598.78 nM	O3 O2 OAZ OAY OAY OAY	Arg439 Arg439 Cme592 Asp633 Ni901 Ni902	NH2 O O OD1 Ni Ni	3.03 2.99 2.37 2.75 2.14 2.67
3-Hydroxythymol-6-O-D-Glucopyranoside (Compound 7)	-6.52	16.69 µM	O3 O4 O2 O4 O4 O2 O2 O6 O5 O6 O4 O4	Arg439 Ala440 Ala440 Kcx490 His492 His492 Asp494 Gly550 Arg609 Asp633 Ni901 Ni902	O O O OQ1 NE2 NE2 OD2 O NH2 OD2 Ni Ni	3.19 2.71 2.72 3.27 2.93 3.04 2.81 2.80 3.15 2.72 3.22 2.78
6- Hydroxythymol-3-O-D-Glucopyranoside (Compound 8)	-6.11	33.31 µM	OAO OAO O6 OAO O4 O2 O3 OAO OAO	Kcx490 His492 Asp494 His519 Cme592 Ala636 Ala636 Ni901 Ni902	OQ1 NE2 OD2 ND1 O O O Ni Ni	3.07 2.72 3.00 3.29 3.32 2.36 3.18 3.18 2.21
Kaempferol-3-O-(6''-O-trans-coumaroyl) glucopyranoside 7-O- 6' coumaroyl glucopyranoside (Compound 6)	-6.11	33.28 µM	OBE OBP OCM	Glu493 His519 His593	OE1 NE2 NE2	2.65 2.64 2.62
Kaempferol 3-Oβ -O-neohesperidoside-7-O-[2-O-(cis-feruloyl)] β -D-glucopyranoside (Coumpound 9)	-1.60	67.38 mM	OAV O6 O6 O6 OBG OBG	Asp494 Gln635 Gly638 Gly638 Ni901 Ni902	OD1 N N O Ni Ni	2.87 2.61 2.91 2.24 2.09 2.75

## Conclusion

In conclusion, a phytochemical analysis of *O. decumbens* was developed to identify new derivatives of kaempferol and thymol, which were not reported previously. Also, the results obtained from the evaluation of different fractions of the plant toward *H. pylori*, various strains of bacteria as well as anti-urease activity of isolated compounds demonstrated satisfactory biological properties of the plant associated with gastrointestinal disease. 

## Conflict of interest

The authors declare that they have no conflict of interest.

## Author contributions

Study concept and design: Mahnaz Khanavi, Mohammad Reza Shams Ardekani and Mahdieh Eftekhari; Phytochemical investigation: Mahdieh Eftekhari, Mahnaz Khanavi; Biological activities*:* Mahdieh Eftekhari, Mohsen Amin, Docking analysis: Mahboubeh Mansourian; Interpretation of data: Mahdieh Eftekhari, Tahmineh Akbarzadeh and Mina Saeedi; Drafting of the manuscript: Mahdieh Eftekhari, Mina Saeedi, Mahnaz Khanavi.
