# Supplementary material for: Anti-Helicobacter pylori Compounds from Oliveria decumbens Vent. through Urease Inhibitory In-vitro and In-silico Studies
Source: Iran J Pharm Res. 2021 Summer;20(3):476–89. doi: 10.22037/ijpr.2021.114485.14876 (PMC8653682; doi:10.22037/ijpr.2021.114485.14876)
Supplement: Supplementary file 1 [file ijpr-20-476-s001.pdf]

Supplementary Materials for

Anti-*Helicobacter pylori* Compounds from *Oliveria decumbens* Vent. through Urease Inhibitory *In-vitro* and *In-silico* Studies

Mahdieh Eftekhari, Mohammad Reza Shams Ardekani, Mohsen Amin, Mahboubeh Mansourian, Mina Saeedi, Tahmineh Akbarzadeh and Mahnaz Khanavi*

*To whom correspondence should be addressed. E-mail: khanavim@tums.ac.ir

Volume 20, Issue 3 (Summer 2021)

This PDF file includes:

Figures S1-S18

(a)

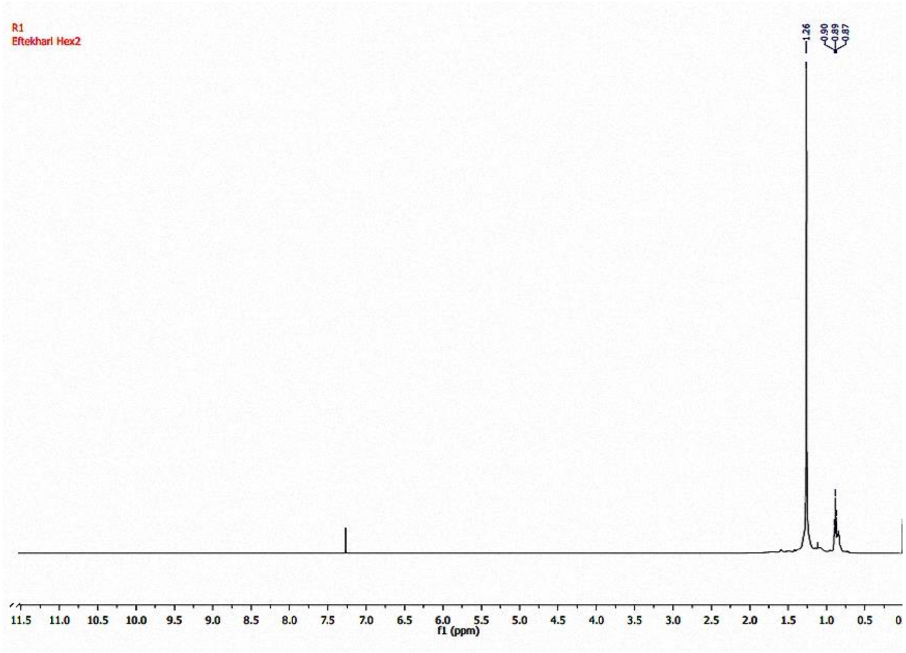

(b)

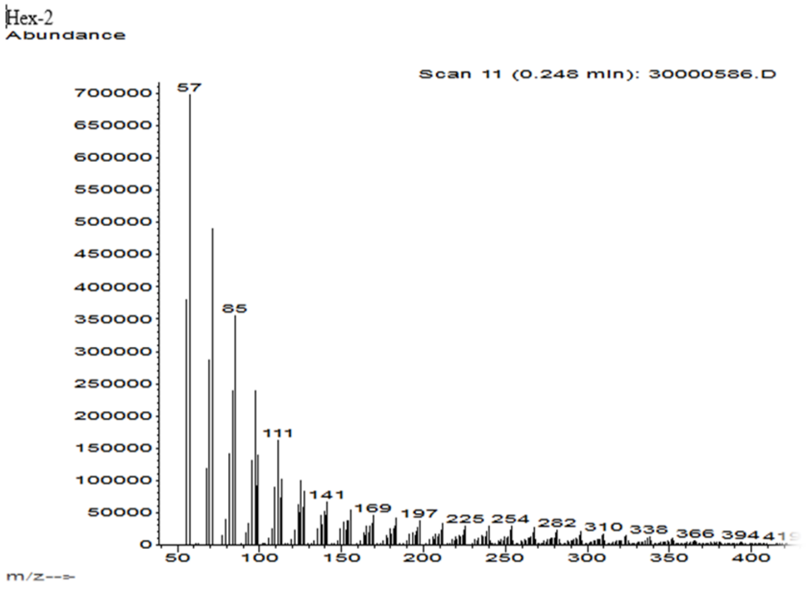

Figure S1. (a) ^{1}HNMR 500 MHz, in $CDCl_3$: Octacosane, (b) EI-MASS :Octacosane

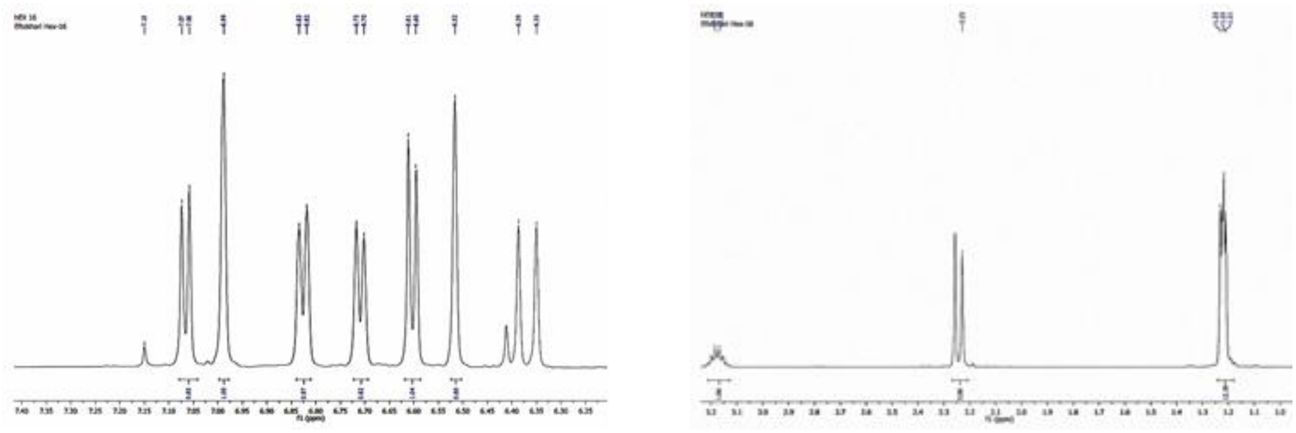

Figure S2. ^{1}HNMR 500 MHz, in $CDCl_3$: Mixture of thymol and car vacrol

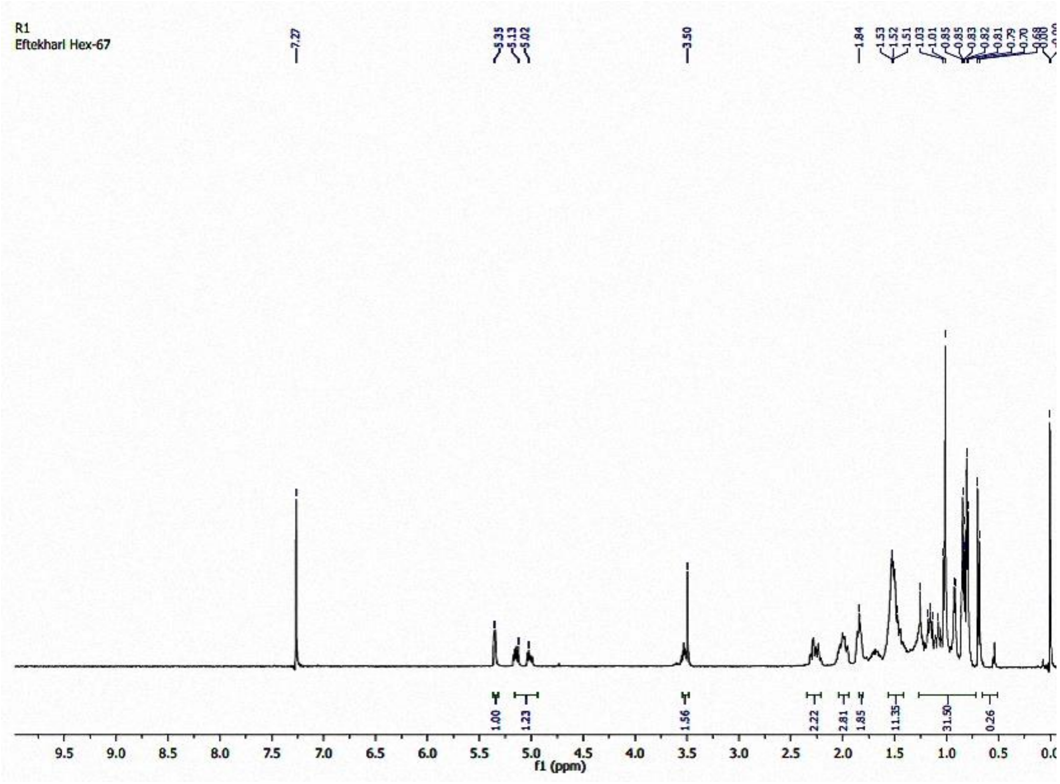

Figure S3.[1] HNMR 500 MHz, in $CDCl_3$: Stigmasterol

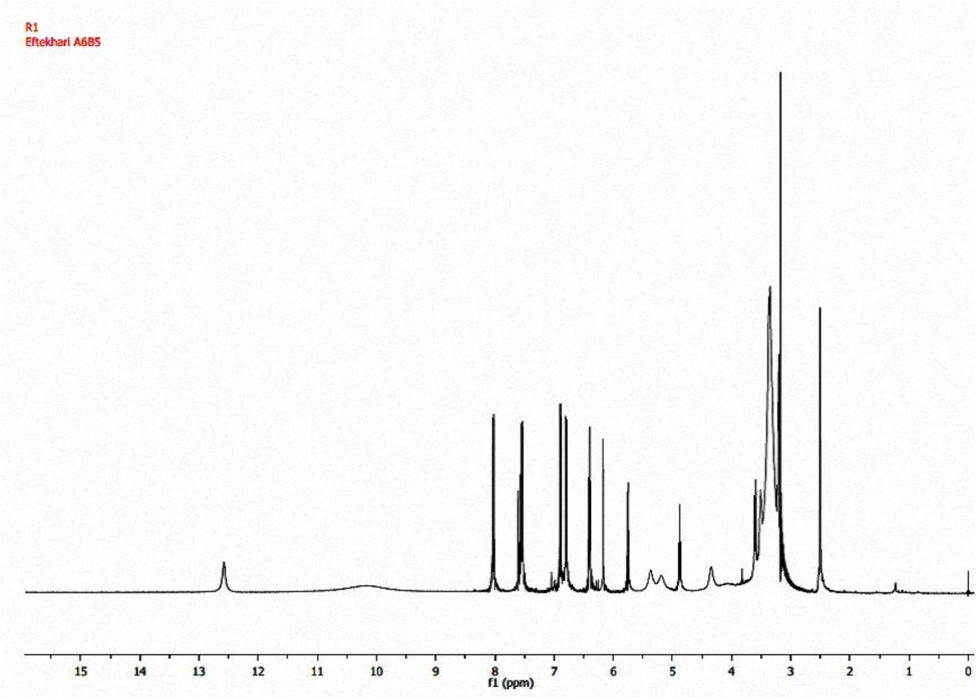

Figure S4. ^{1}HNMR 500 MHz, in DMSO-d_{6}: Kaempferol-3-O-(6''-O-trans-coumaryl)glucoside

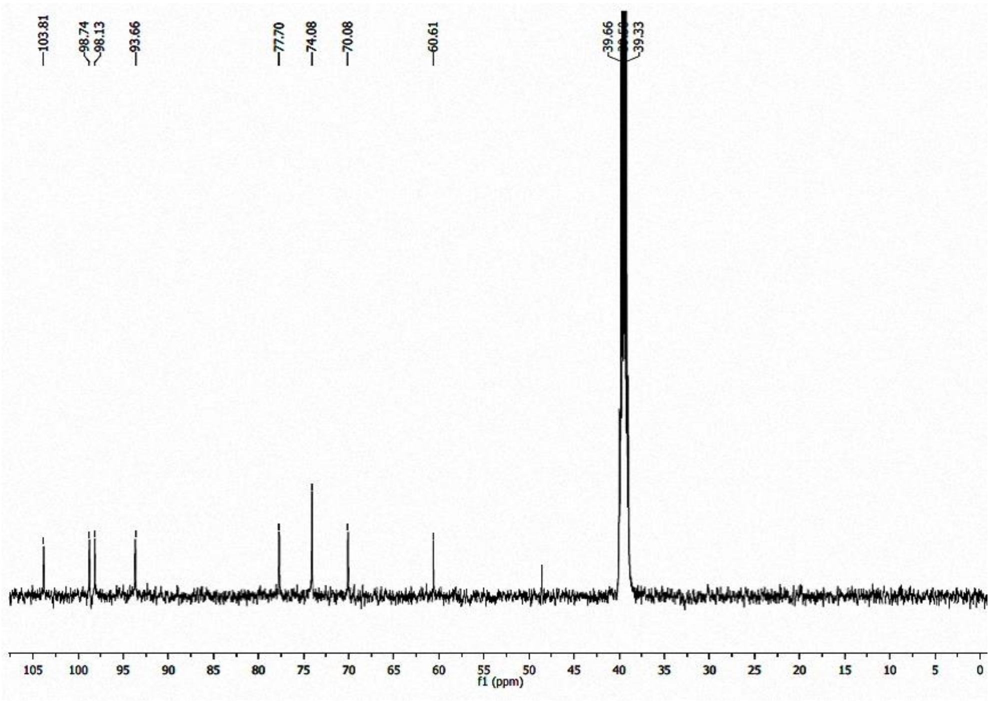

Figure S5. ^{13}CNMR 125 MHz, in DMSO-d_6: Kaempferol-3-O-(6"-O-trans-coumaryl)glucoside

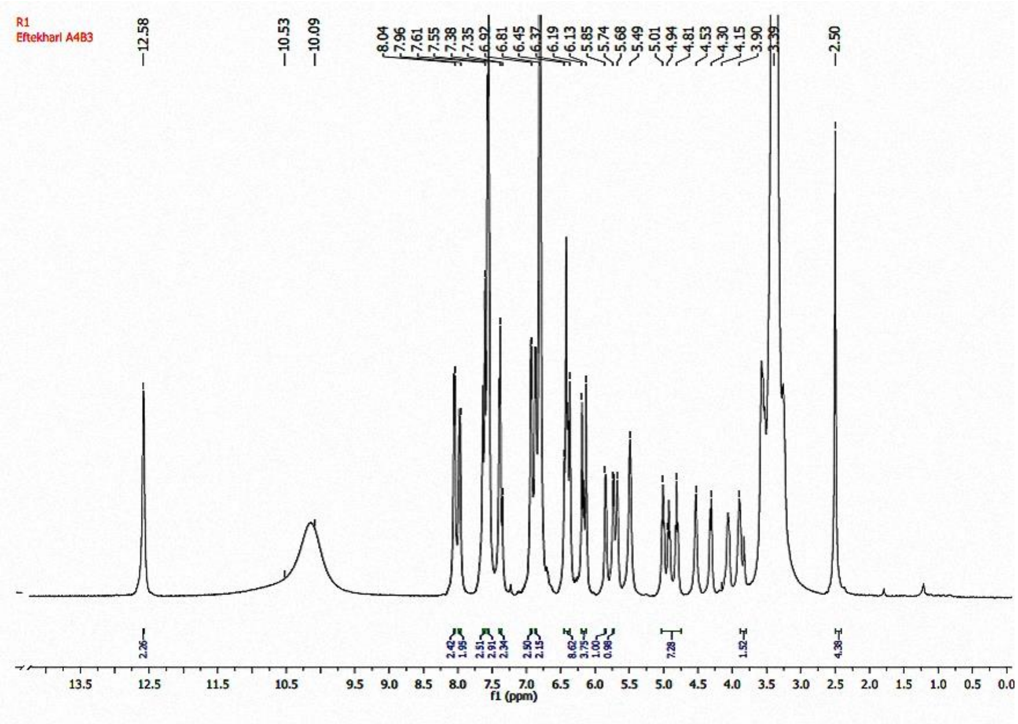

Figure S6. ^{1}HNMR 500 MHz, in DMSO-d_6: Kaempferol 3 -O-(6’’-O-trans-coumaryl)glucoside 7-O-(6’’’-O-trans-coumaryl) glucoside.

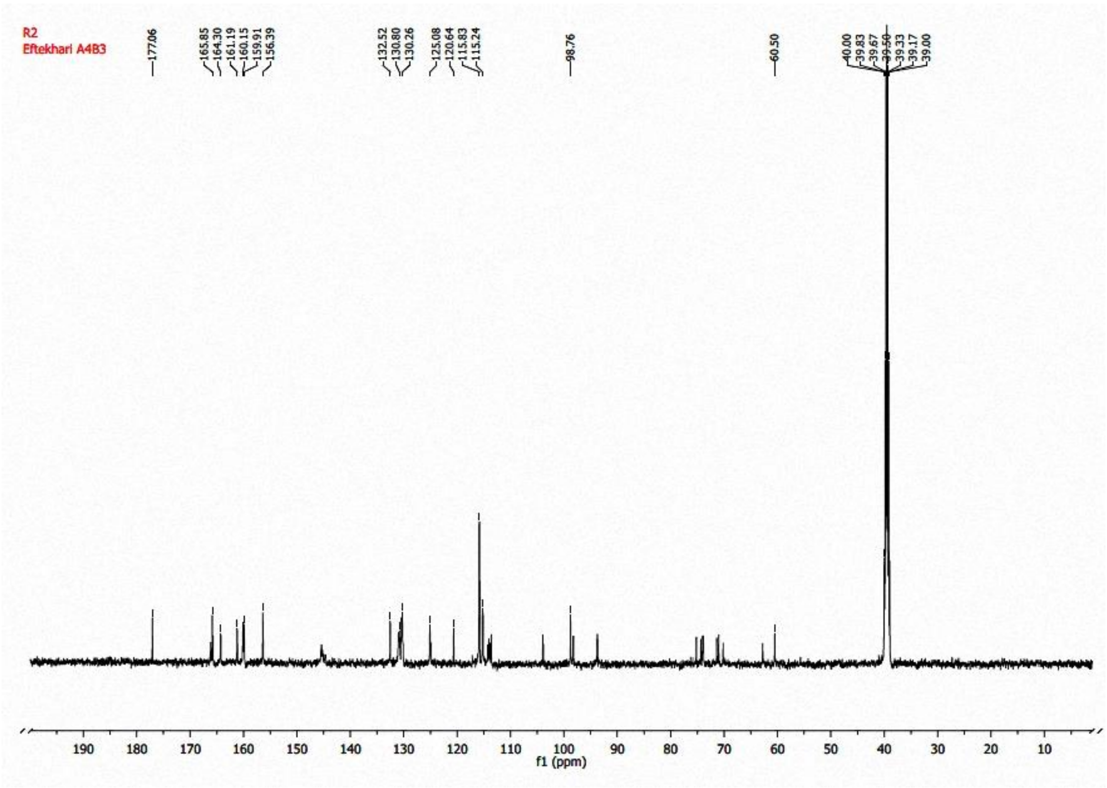

Figure S7. ^{13}CNMR 125, in DMSO-d_6, Kaempferol 3 -O-(6’’-O-trans-coumaryl)glucoside 7-O-(6’’’-O-trans-coumaryl) glucoside.

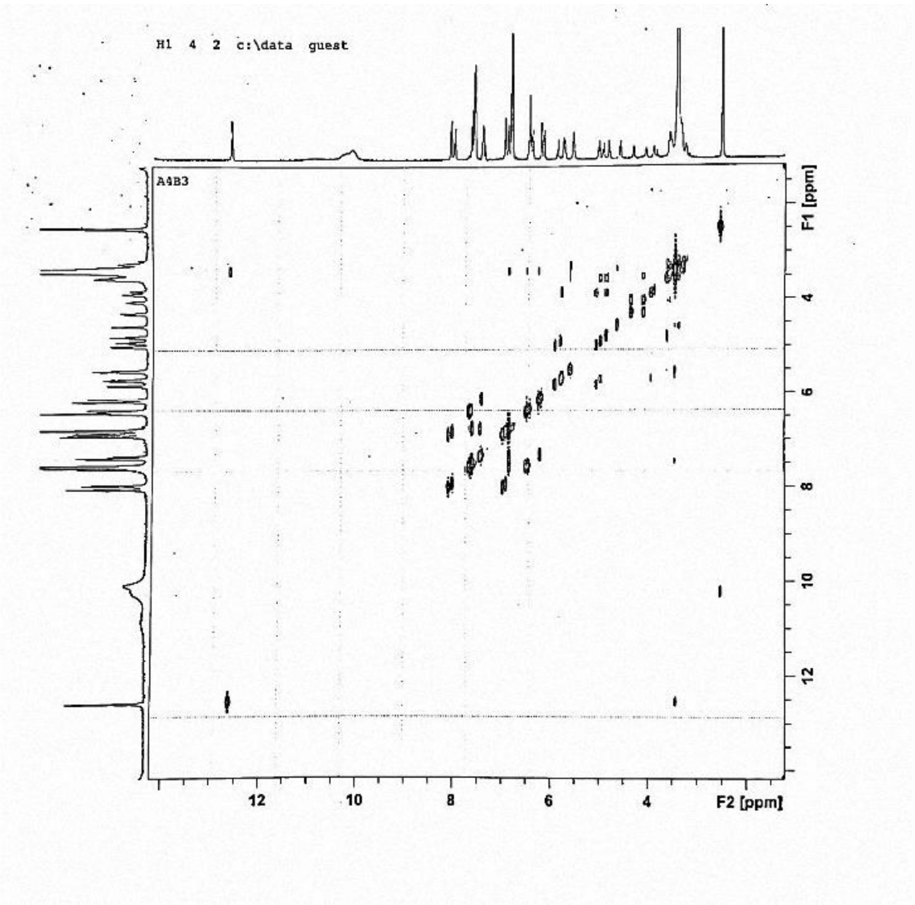

Figure S8. COSY, in DMSO-d$_6$, Kaempferol 3 -O-(6''-O-trans-coumaryl)glucoside 7-O-(6'''-O-trans-coumaryl) glucoside

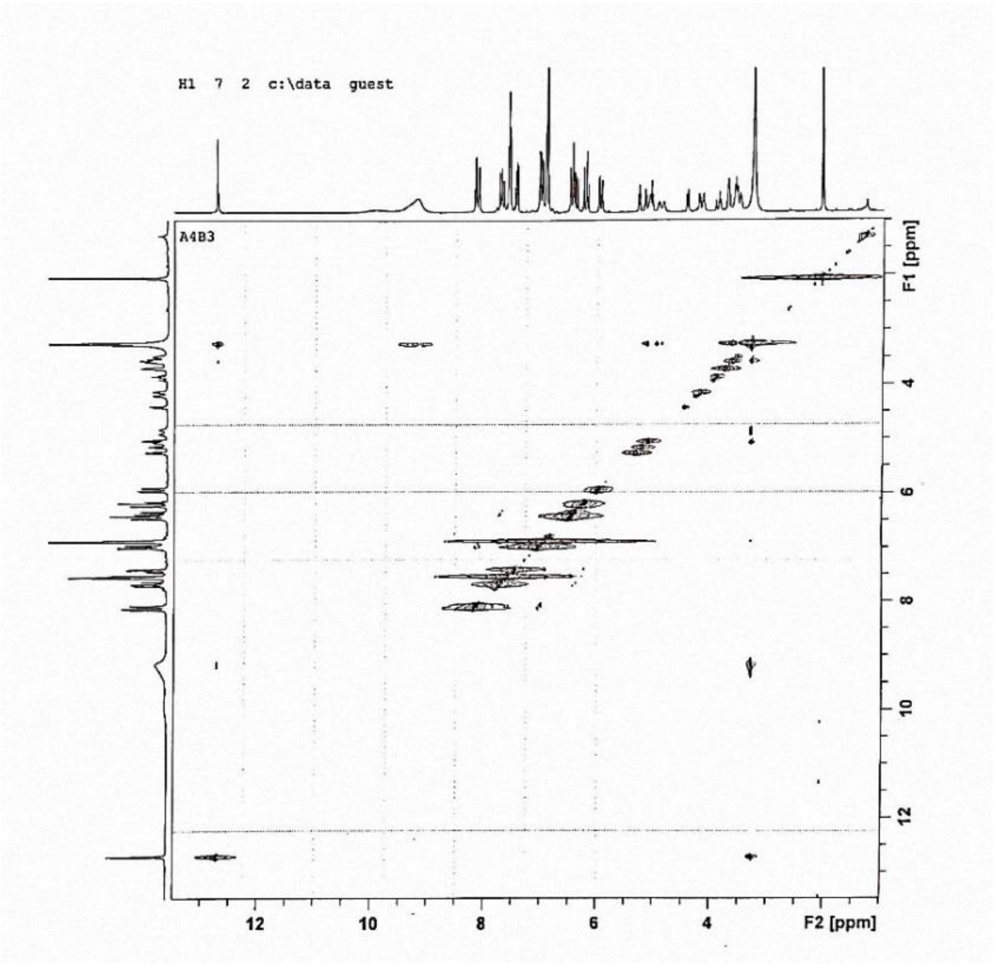

Figure S9. NOSEY, in Acetone-d_6, Kaempferol 3 -O-(6’’-O-trans-coumaryl)glucoside 7-O-(6’’’-O-trans-coumaryl) glucoside

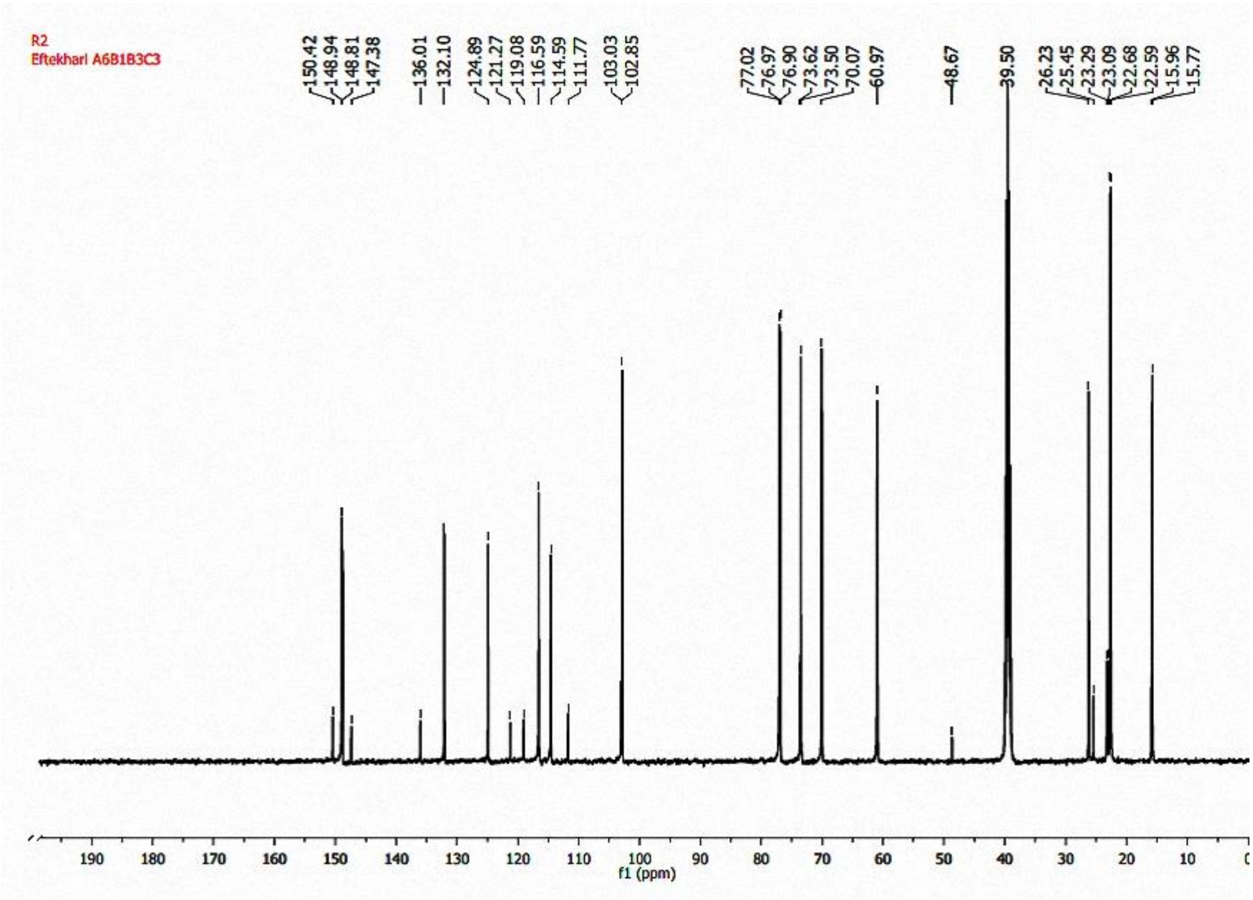

Figure S10. ^{13}CNMR 125 MHz, in DMSO-d_6: 3-Hydroxythymol-6-O-D-Glucopyranoside- &6-Hydroxythymol-3-O-D-Glucopyranoside

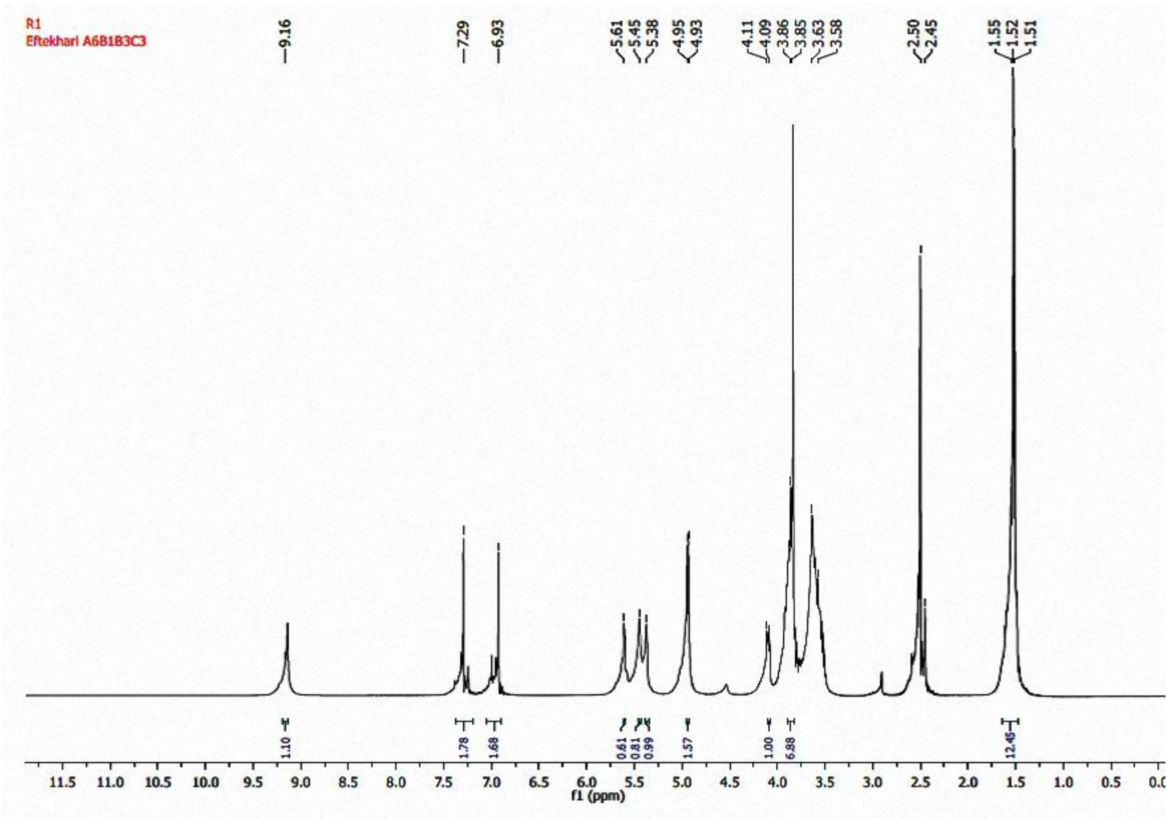

Figure S11. ^{1}HNMR 500 MHz, in DMSO-d_6, 3-Hydroxythymol-6-O-D-Glucopyranoside- &6-Hydroxythymol-3-O-D-Glucopyranoside

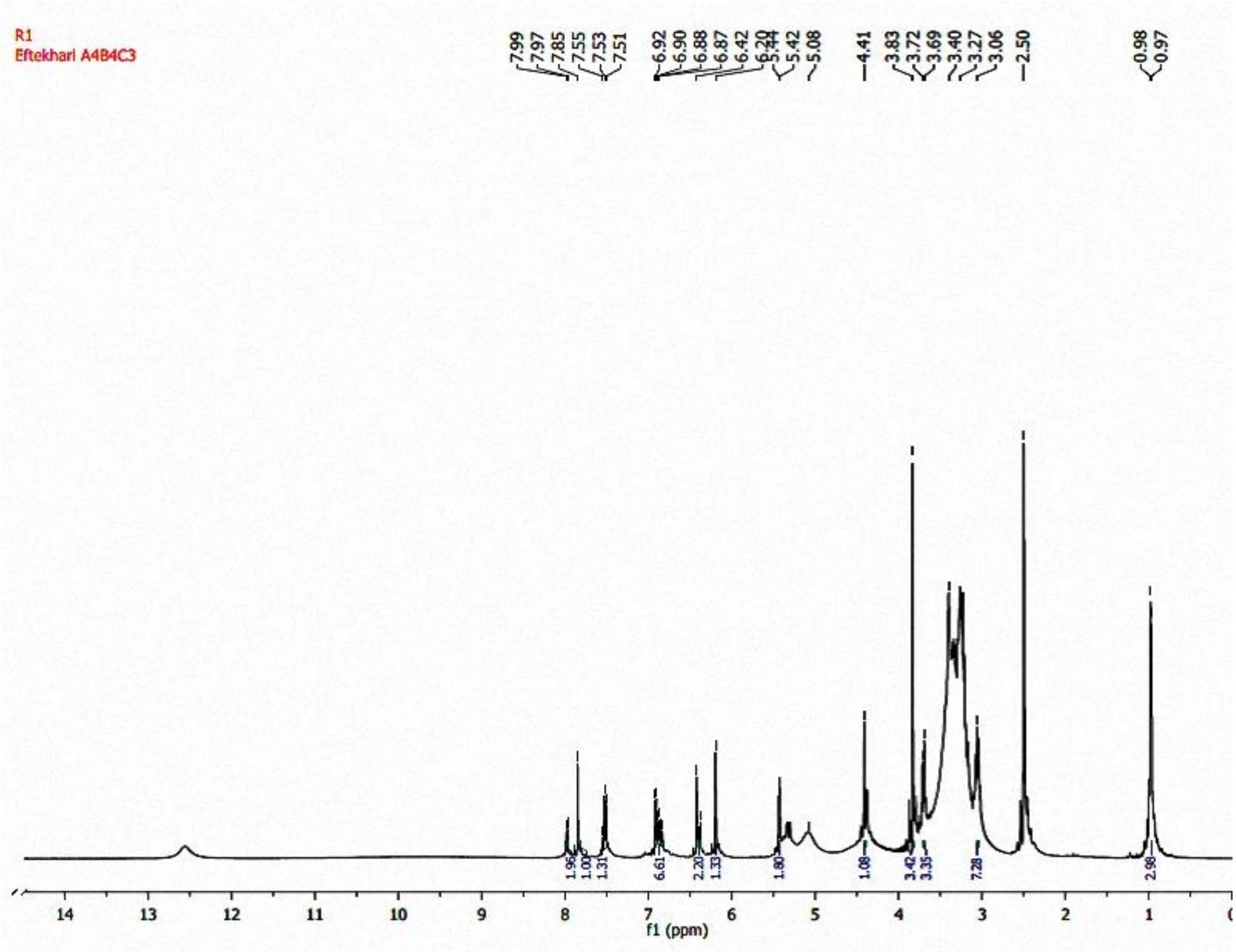

Figure S12. ^{1}HNMR 500 MHz, in DMSO-d$_6$: : kaempferol 3-O-neohesperidoside-7-O-[2-O-(cis-feruloyl)]-D-glucopyranoside

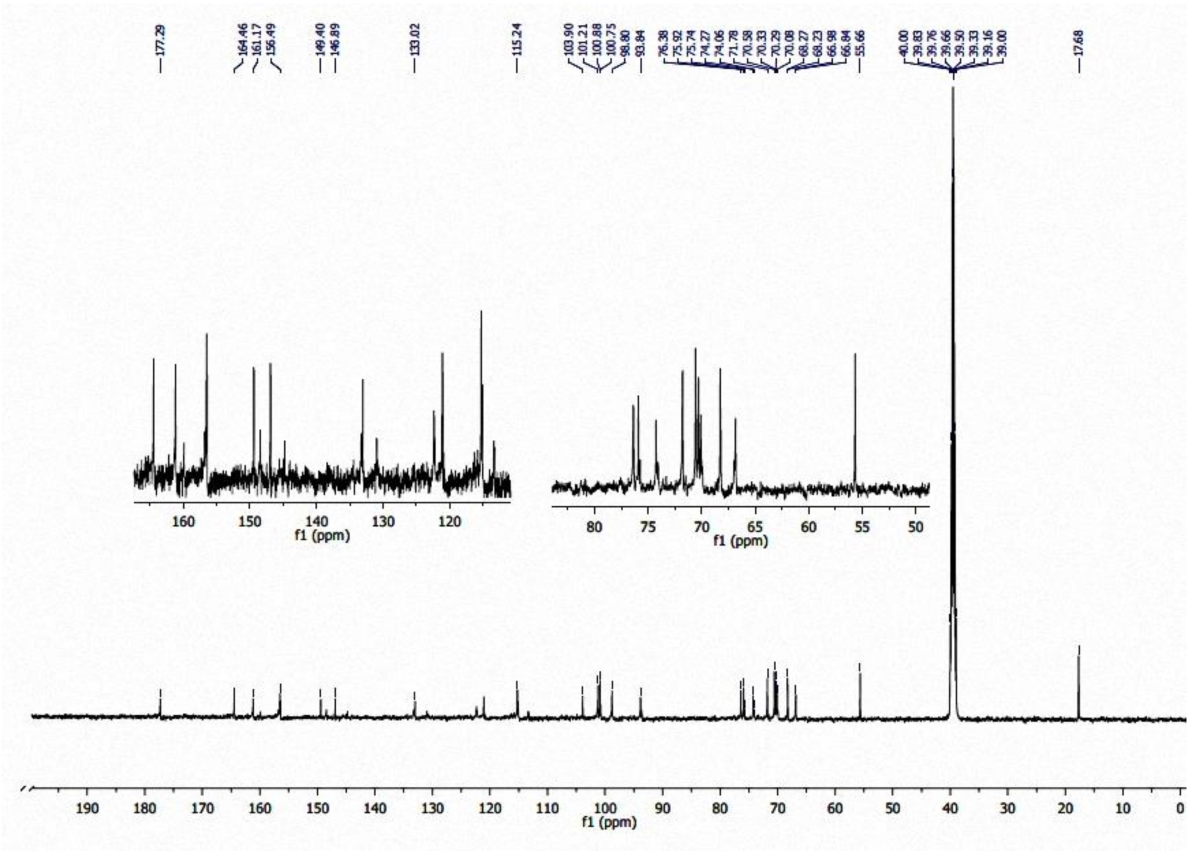

Figure S13. ^{13}CNMR 125 MHz, in DMSO-d_6:: kaempferol 3-O-neohesperidoside-7-O-[2-O-(cis-feruloyl)]-D-glucopyranoside

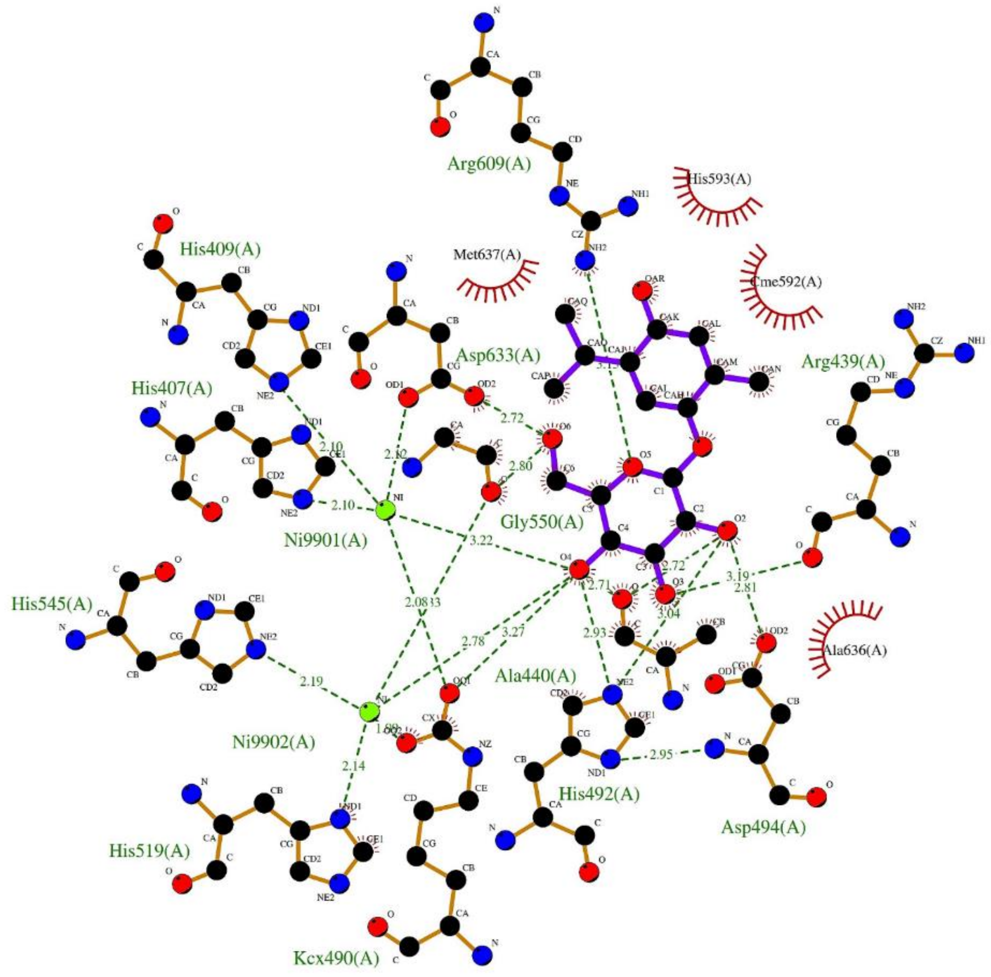

Figure S14. Schematic interaction of the best docking resulting from AutoDock software presented by LigPlot software for thymol. In this figure, the compound exposure is highlighted in blue. Hydrogen bonding is in green and van der Waals interactions are in red circulars.

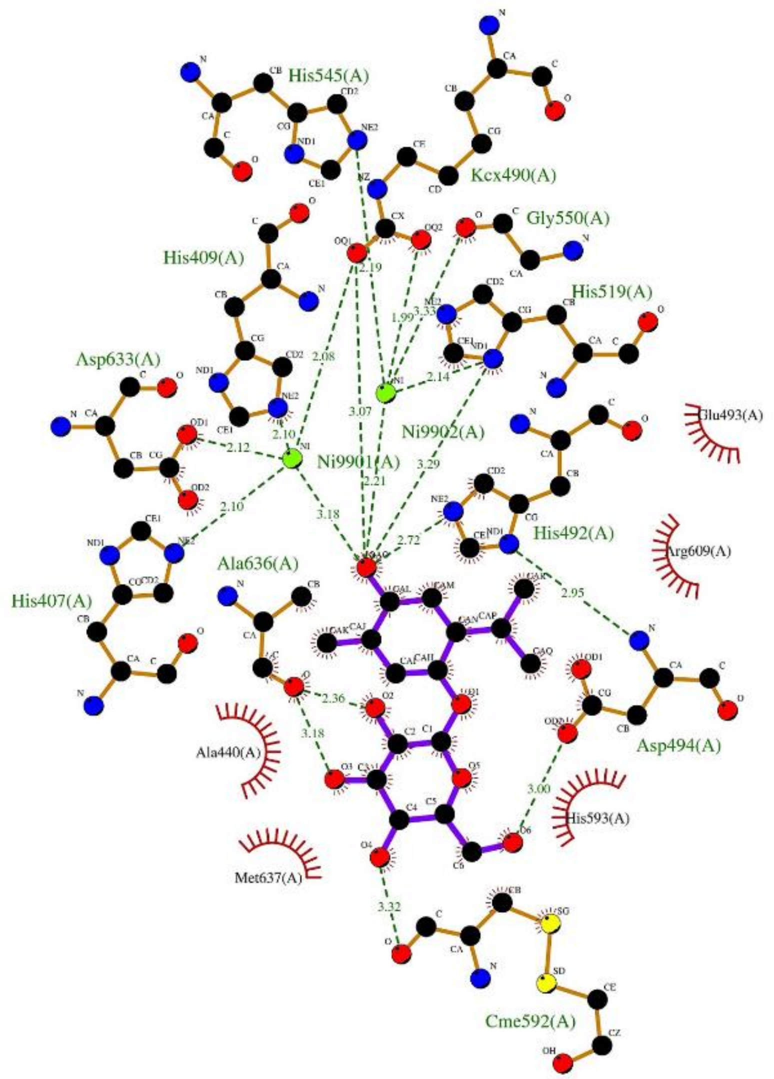

Figure S15. Schematic interaction of the best docking resulting from AutoDock software presented by LigPlot software for compound 7. In this figure, the compound exposure is highlighted in blue. Hydrogen bonding is in green and van der Waals interactions are in red circulars.

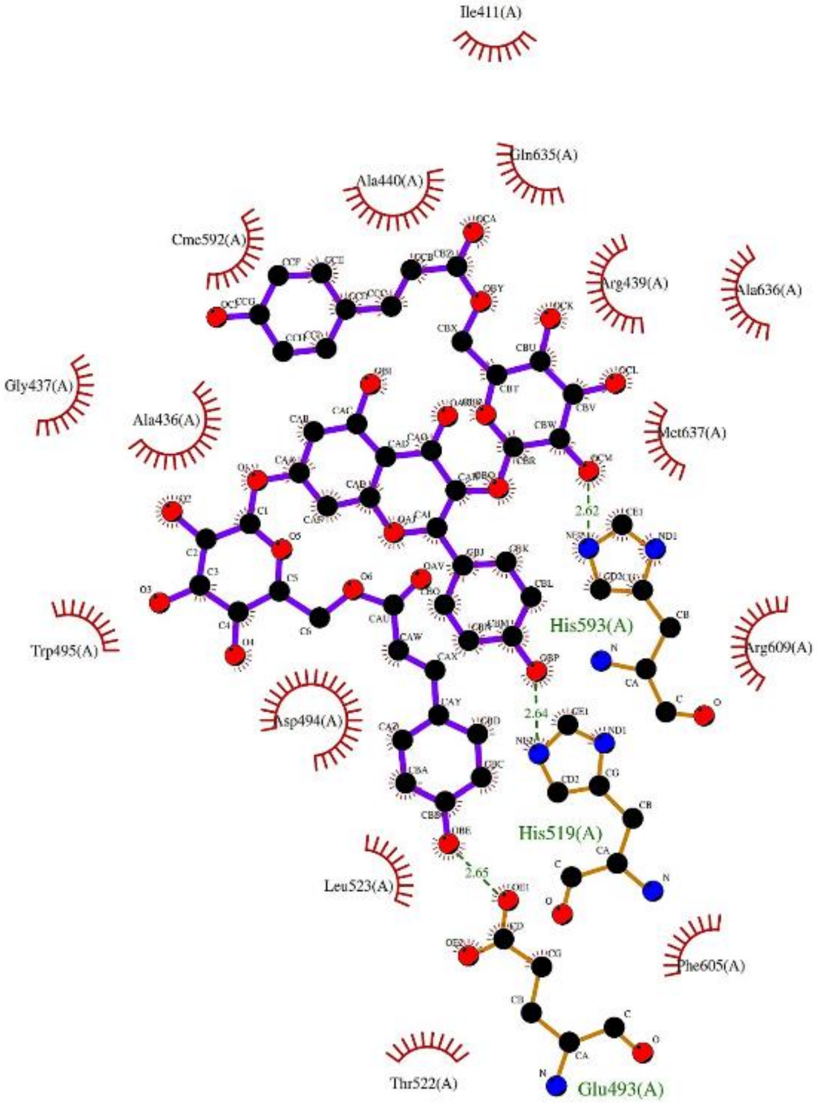

Figure S16. Schematic interaction of the best docking resulting from AutoDock software presented by LigPlot software for compound 8. In this figure, the compound exposure is highlighted in blue. Hydrogen bonding is in green and van der Waals interactions are in red circulars.

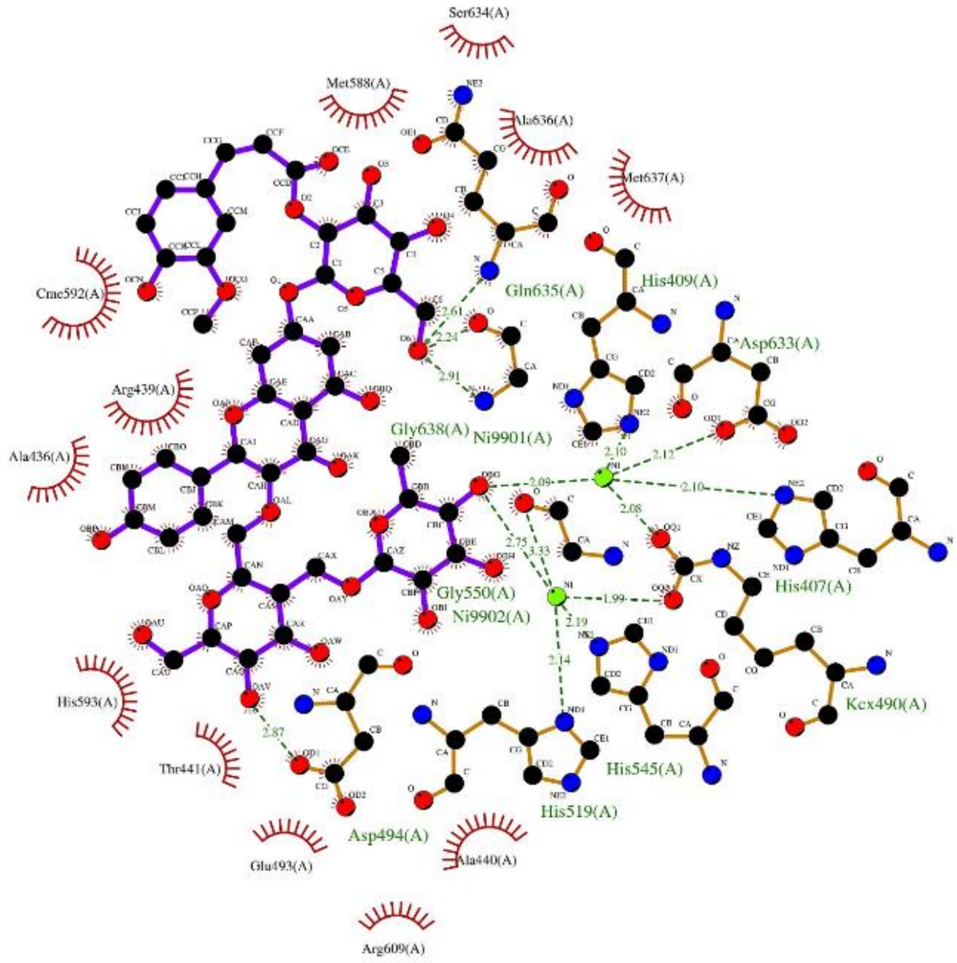

Figure S17. Schematic interaction of the best docking resulting from AutoDock software presented by LigPlot software for compound 6. In this figure, the compound exposure is highlighted in blue. Hydrogen bonding is in green and van der Waals interactions are in red circulars.

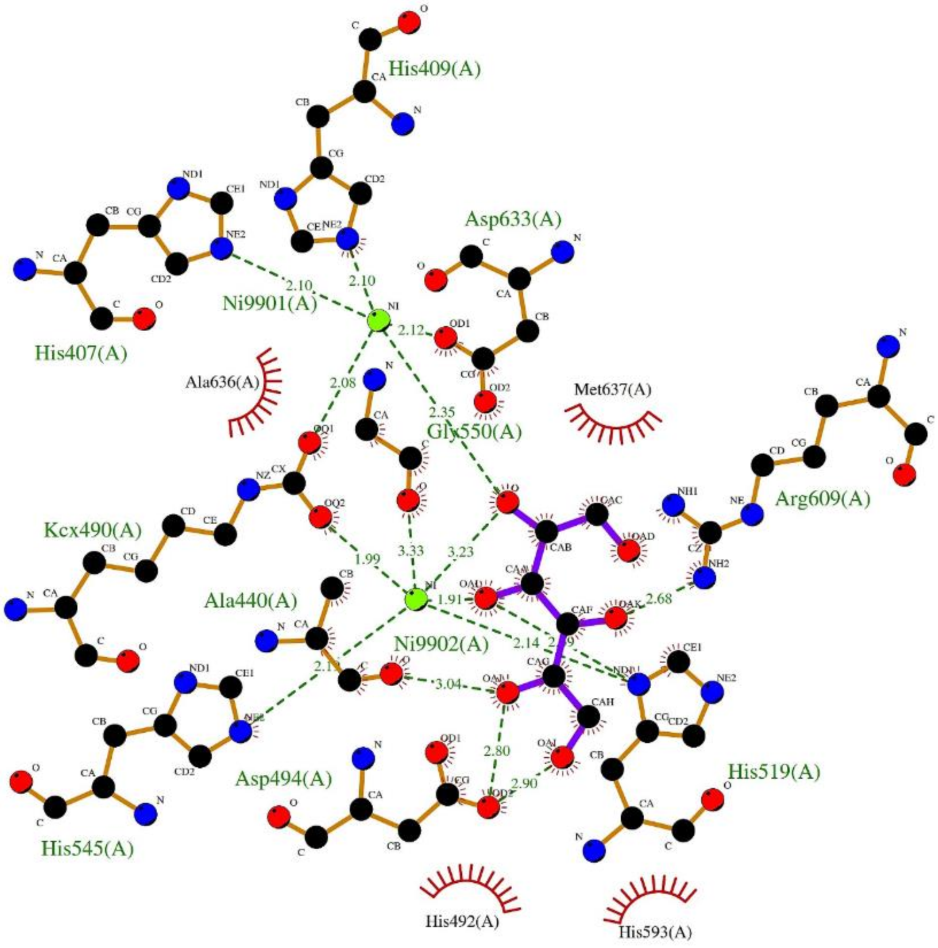

Figure S18. Schematic interaction of the best docking resulting from AutoDock software presented by LigPlot software for compound 9. In this figure, the compound exposure is highlighted in blue. Hydrogen bonding is in green and van der Waals interactions are in red circulars.
